# A Förster Resonance Energy Transfer (FRET)-Based Immune Assay for the Detection of Microcystin-LR in Drinking Water

**DOI:** 10.3390/s24103204

**Published:** 2024-05-17

**Authors:** Alessandro Capo, Angela Pennacchio, Concetta Montagnese, Antonis Hadjiantonis, Panayiota Demosthenous, Alessandro Giusti, Maria Staiano, Sabato D’Auria, Antonio Varriale

**Affiliations:** 1 Istituto di Scienze dell’Alimentazione, CNR, URT-Napoli, 80100 Napoli, Italy; alessandro.capo@cnr.it (A.C.); antonio.varriale@cnr.it (A.V.); 2 Istituto di Scienze dell’Alimentazione, CNR, 83100 Avellino, Italy; angela.pennacchio@cnr.it (A.P.); concetta.montagnese@cnr.it (C.M.); maria.staiano@cnr.it (M.S.); 3 CY.R.I.C Cyprus Research and Innovation Center Ltd., Egomi, 2414 Nicosia, Cyprus; antonish@cyric.eu (A.H.); p.demosthenous@cyric.eu (P.D.); alessandro@cyric.eu (A.G.); 4 Dipartimento di Scienze Bio-Agroalimentari, CNR, 00185 Roma, Italy

**Keywords:** biosensors, *Cyanobacteria bloom*, microcystin, FRET, fluorescence spectroscopy

## Abstract

*Cyanobacteria bloom* is the term used to describe an abnormal and rapid growth of cyanobacteria in aquatic ecosystems such as lakes, rivers, and oceans as a consequence of anthropic factors, ecosystem degradation, or climate change. Cyanobacteria belonging to the genera *Microcystis*, *Anabaena*, *Planktothrix*, and *Nostoc* produce and release toxins called microcystins (MCs) into the water. MCs can have severe effects on human and animal health following their ingestion and inhalation. The MC structure is composed of a constant region (composed of five amino acid residues) and a variable region (composed of two amino acid residues). When the MC variable region is composed of arginine and leucine, it is named MC-LR. The most-common methods used to detect the presence of MC-LR in water are chromatographic-based methods (HPLC, LC/MS, GC/MS) and immunological-based methods (ELISA). In this work, we developed a new competitive Förster resonance energy transfer (FRET) assay to detect the presence of traces of MC-LR in water. Monoclonal antibody anti-MC-LR and MC-LR conjugated with bovine serum albumin (BSA) were labeled with the near-infrared fluorophores CF568 and CF647, respectively. Steady-state fluorescence measurements were performed to investigate the energy transfer process between anti-MC-LR 568 and MC-LR BSA 647 upon their interaction. Since the presence of unlabeled MC-LR competes with the labeled one, a lower efficiency of FRET process can be observed in the presence of an increasing amount of unlabeled MC-LR. The limit of detection (LoD) of the FRET assay is found to be 0.245 nM (0.245 µg/L). This value is lower than the provisional limit established by the World Health Organization (WHO) for quantifying the presence of MC-LR in drinking water.

## 1. Introduction

*Cyanobacteria* are part of most aquatic ecosystems, including lakes, rivers, and oceans [1]. In particular conditions, uncontrolled and rapid growth of these bacteria occurs. This phenomenon is called “*Cyanobacteria bloom*” (or blue-green algae). Various external factors are responsible for it, such as nutrient over-enrichment of water (anthropic factor), ecosystem degradation [1], an increase in water temperature, and an increase in the CO_2_ level in the atmosphere [2,3].

The *Cyanobacteria* belonging to the genera *Microcystis, Anabaena, Planktothrix*, and *Nostoc* produce toxic secondary metabolites called microcystins (MCs), a group of toxins (cyanotoxins) that have hepatotoxic, neurotoxic, and dermatoxic effects on animals and humans.

From a structural point of view, MCs are cyclic heptapeptides. They are always composed of the following five amino acid residues: (1) β-amino acid (ADDA); (2) alanine (D-ala); (3) D-β-methyl-isoaspartate (D-β-Me-isoAsp); (4) N-methyl-dehydro-alanine (Mdha); and (5) glutamic acid (D-Glu), plus two additional variable amino acid residues. The variable portion of MCs is responsible for microcystin variability and the different degree of toxicity [4,5]. If the MC variable region is composed of arginine (R) and leucine (L), the microcystin is named microcystin-LR (MC-LR).

The International Agency for Research on Cancer (IARC) has classified MC-LR as potential human carcinogens (group 2B) [6], and the World Health Organization (WHO) has fixed a provisional maximum-allowed level of MC-LR in drinking water at 1.0 μg/L and a daily intake value (TDI) of 0.04 μg/kg MC-LR/body weight [7]. In the European Union, the regulations for limiting values of cyanotoxins in drinking water at the moment are not well-defined due to incomplete chronic toxicity data [8].

The most common methods used to detect the presence of MC-LR are chromatographic-based methods (HPLC, LC/MS, GC/MS), immunological-based methods (ELISA), and protein phosphatase inhibition assays (PPIA) [9]. These methods allow for qualitative and quantitative analyses, even if their applications are often restricted due to the complex and time-consuming extraction procedure, the presence of interferences, and the cross-reactivity of congeners [10].

Recently, different biosensor approaches have been developed to complement the analytical methods to detect the presence of MC-LR and related congeners. These methods are based on the use of antibodies coupled with optical or electrochemical techniques [11,12]. Förster resonance energy transfer (FRET) is a powerful tool with which to study molecular interactions, characterize molecular conformation variations in bio/molecules, and design advanced optical biosensors [13,14].

FRET is a mechanism that involves the presence of at least of two chromophore molecules: the donor molecule and the acceptor molecule. The donor molecule in the electronic excited state may transfer energy to the acceptor molecule via a non-radiative dipole–dipole coupling process. The distance between donor and acceptor molecules is essential. In fact, the efficiency of the energy transfer between the donor and acceptor molecules is inversely proportional to the sixth power of their distance. An additional essential FRET requirement is that the emission spectrum of the donor molecule must partially overlap with the absorption spectrum of the acceptor molecule [15]. The possibility to use FRET methodology applied to biosensor design greatly reduced the analysis time, making it very attractive for the development of new assays [13].

In this work, we developed a Förster resonance energy transfer (FRET)-based immune assay using a specific monoclonal antibody to detect the presence of traces of microcystin-LR in water.

For this purpose, we labeled the monoclonal antibody anti-microcystin-LR with the fluorescence probe CF568 (it works as energy donor) and the conjugate MC-LR with bovine serum albumin (BSA) with the fluorescence probe CF647 (it works as an energy acceptor).

The formation of the complex between anti-MC-LR 568 and MC-LR 647 in the absence of and the presence of MC-LR was investigated as a variation in the FRET efficiency.

The results show that this assay can detect the presence of MC-LR with a LoD of 0.245 nM (0.245 µg/L).

## 2. Materials and Methods

### 2.1. Materials

MC-LR mouse monoclonal antibody (clone MC10E7; ref. #ALX-804-320-C200) and Microcystin-LR (ref. # ALX-350-012-M001) were purchased from Enzo Life Sciences (Enzo Life Sciences Inc., Farmingdale, NY, USA). The Microcystin-LR BSA (MC-LR BSA) conjugate (ref. # DAGHY010) was purchased from Creative Diagnostics (Creative Diagnostics, Shirley, NY, USA), while N-hydroxysuccinimide (NHS), N-(3-dimethylaminopropyl)-N’-ethylcarbodiimide hydrochloride (EDC), and ethylenediamine were purchased from Sigma-Aldrich (Sigma-Aldrich S.r.l., Milan, Italy). CF568 and CF647 fluorescent probes were purchased from Biotium (Biotium Inc. Fremont, CA, USA). Microplates (96-well), C8 lockwell maxisorp Nunc (ref. cod. 446469), was purchased from Thermo Fisher Scientific Inc. (Waltham, MA, USA). Goat polyclonal IgG–HRP secondary antibody used in the ELISA experiments was purchased from Abcam (Abcam plc. Oregon, OR, USA). The Sephadex G75 chromatographic resin was purchased from Sigma-Aldrich. All other chemicals were commercial samples of the purest quality.

### 2.2. Indirect ELISA Test

The binding capability of the anti-microcystin-LR (anti-MC-LR) was investigated by indirect ELISA test. In brief, a solution of 50 μL of the conjugate microcystin-LR BSA (MC-LR BSA) (from 0.008 mg/mL to 0.13 mg/mL) diluted in the coating buffer at pH 9.6 was added to individual wells and incubated overnight at 4 °C. After overnight incubation, the following steps were performed: the wells were washed three times with a solution of 200 μL TBS at pH 7.4 for 10 min; then, they were incubated with a 100 μL blocking solution (TBS, 5% skim milk) for 1 h at 37 °C.

After this incubation, the wells were washed three times with a solution of 200 μL TBS-0.05% Tween 20. A solution of anti-MC-LR at 1.0 μg/mL (50 μL) was added to each well followed by incubation at 37 °C for 1 h. The wells were washed three-times with a solution of 200 μL TBS-0.05% Tween 20 at pH 7.4 for 10 min, then a solution of 50 μL of diluted anti-mouse secondary antibody, at a concentration of 1 μg/mL, was added to each well and incubated at 37 °C for 1 h. A solution of 100 μL of the 3,3′, 5,5′-tetramethylbenzidine (TMB) per individual well was added and incubated at RT for 10 min. Finally, a 100 μL stop solution was added to the wells (2.5 M HCl), and the absorbance at 450 nm was measured by Tecan Infinite 200 PRO plate reader (Tecan Group Ltd., Männedorf, Switzerland).

A similar protocol was used to characterize the binding capability of the labeled anti-MC-LR.

### 2.3. Preparation of the Labeled Antibody Anti-Microcystin-LR

The anti-MC-LR was labeled with the fluorescence probe Biotium CF568, selected as the energy donor dye for the FRET assay. Briefly, 400 µg of lyophilized anti-MC-LR were re-suspended in a solution of 100 µL of 0.1 M sodium hydrogen carbonate buffer at pH 8.3 to a final concentration of 4.0 mg/mL. One µmole of Biotium CF568 was re-suspended in a solution of 100 µL dimethyl sulfoxide (DMSO) to a final concentration of 10 mg/mL. The reaction mix was prepared by adding 2 µL of Biotium CF568 to 98 µL of the anti-MC-LR (molar dye–antibody ratio: 1:12), and it was incubated in the dark under mild stirring conditions for 1 h at room temperature. At the end of the incubation, the solution was loaded on a Sephadex G75 column to separate the labeled antibody molecules from the unreacted dye (in excess). The chromatography step was performed by using a solution of 20 mM sodium phosphate 150 mM NaCl pH 7.4 buffer. The collected fractions (100 µL each) were analyzed by the spectrophotometer Jasco V-550 (wavelength range 220–700 nm) using a TrayCell^®^ cuvette (Hellma GMBH and CO. KG, Mullheim, DE, Germany). The fractions with peaks at 280 nm and 562 nm (these two absorbance values are characteristic of the labeled Ab with CF568) were pooled, filtered, and concentrated by a Vivaspin^®^ 500 (Sartorius AG, Göttingen, Germany) centrifugal concentrators system with a 10 kDa cut-off up to 250 µL.

### 2.4. Preparation of the MC-LR BSA Labeling

MC-LR BSA was labeled with the fluorescent probe Biotium CF647 selected as the energy acceptor dye for the FRET assay. One mg of lyophilized MC-LR BSA was re-suspended in a solution of 144 µL of 0.1 M sodium hydrogen carbonate pH 8.3 buffer, to a final concentration of 6.9 mg/mL. One µmole of Biotium CF647 was re-suspended in a solution of 100 µL DMSO to a final concentration of 10 mg/mL (molar dye–antibody ratio: 12:1). A solution of 16 µL of Biotium CF647 was added to a solution of 144 µL of the MC-LR BSA. The reaction was conducted in the dark under mild stirring conditions for 1 h at room temperature. After 1 h, the reaction mix was immediately stopped, and the solution was loaded on a Sephadex G75 column to separate the labeled molecules from the unreacted dye (in excess). The chromatography step was performed by using a solution of 20 mM sodium phosphate 150 mM NaCl pH 7.4 buffer. The collected fractions (100 µL each) were analyzed by the spectrophotometer Jasco V-550 (wavelength range 220 nm–700 nm) using a TrayCell^®^ cuvette (Hellma GMBH and CO. KG, Mullheim, DE, Germany). The fractions with a peak at 280 nm and 647 nm (related to CF647™ dye) were pooled, filtered, and concentrated by a Vivaspin^®^ 500 (Sartorius AG, Göttingen, Germany) centrifugal concentrators system with a 10 kDa cut-off up to 250 µL.

### 2.5. Characterization of Anti-MC-LR 568 and MC-LR BSA 647 Conjugates

To evaluate the purity of the anti-MC-LR 568 and the MC-LR BSA 647, a sodium-dodecyl sulfate–polyacrylamide gel electrophoresis (15% SDS-PAGE) was performed.

The two labeled molecules (donor and acceptor) were characterized by absorption spectroscopy experiments carried out on a spectrophotometer Jasco V-550 (wavelength range 220 nm–700 nm) by using a TrayCell^®^ cuvette (Hellma GMBH and CO. KG, Mullheim, DE, Germany).

The molar concentration of the two labeled molecules, the yield of the labeling reactions (%), and the degree of labeling (DOL) for the two molecules were calculated by the following equations:

(1) $$[\mathrm{Conjugate}]\;M=\frac{A_{280}-(A_{max}\times CF)}{ɛ}\times \mathrm{dilution}\;\mathrm{factor},$$

[Conjugate] mg/mL = [Conjugate] M × MW,(2)

Total amount of conjugate (mg) = [Conjugate] mg/mL × Volume (mL),(3)

(4) $$\mathrm{Yield}\;\mathrm{of}\;\mathrm{reaction}\;(\%)=\frac{Total\;amount\;of\;Conjugate\;(mg)}{Initial\;amount\;of\;protein\;(mg)}\times 100,$$

(5) $$\mathrm{DOL}=\frac{A_{max}\times MW\times dilution\;factor}{{ɛ}^{\prime }\times [Conjugate]\;mg/mL},$$

where:

- ε = molar absorption coefficient of anti-MC-LR = 210,000 M^−1^ cm^−1^ and MC-LR BSA = 43,824 M^−1^ cm^−1^;
- A_280_ and A_max_ = the absorbance of the labeled molecules at 280 nm and the absorption maximum, respectively;
- CF = absorbance correction factor (CF568 = 0.08; CF647 = 0.03);
- Dilution factor = the extent to which the labeled molecules were diluted for absorbance measurement;
- MW = molecular weight (anti-MC-LR = 150,000 Da; MC-LR BSA = 67,463 Da);
- ε′ = molar absorption coefficient of the dye (CF568 = 100,000 M^−1^ cm^−1^; CF647 = 240,000 M^−1^ cm^−1^).

### 2.6. Fluorescence Steady-State Measurements

Steady-state fluorescence emission experiments were recorded on a FP8600 Jasco fluorometer with a cell temperature-controlled sample holder. The anti-MC-LR 568 and the MC-LR BSA 647 were diluted at values of 0.06 O.D. at their maximum absorbance values (565 nm for the donor and 650 nm for the acceptor) to avoid the inner filter effect [16,17]. Anti-MC-LR 568 and MC-LR BSA 647 were excited at 565 nm and 650 nm, respectively. Emission spectra were recorded from 570 nm to 750 nm (anti-MC-LR 568) and from 655 nm to 800 nm (MC-LR BSA 647) at 1.0 nm intervals at a scan speed of 100 nm/min using a 1.0 cm light path fluorescence quartz cuvette. Both excitation and emission slit widths were fixed at 5 nm. All measurements were carried out at 25 °C with an accuracy of ±0.5 °C in PBS buffer, pH 7.4, in a total volume of 500 μL [18]. The FRET phenomenon associated with the binding event between the anti-MC-LR 568 and MC-LR BSA 647 was investigated by steady-state fluorescence spectroscopy measurements. Titration experiments were carried out with a fixed concentration of anti-MC-LR 568 (100 nM) and increasing concentrations of MC-LR BSA 647 (0–100 nM) to reach the plateau of the fluorescence emission. For each concentration of MC-LR BSA 647 tested, the samples were incubated for 30 min at 25 °C. The buffer was used as blank, and its emission contribution was subtracted from the spectra.

### 2.7. Microcystin FRET Competitive Assay Development

The competitive FRET assay was carried out at a fixed concentration of anti-MC-LR 568 (50 nM) and with a fixed concentration of MC-LR BSA 647 (100 nM) in the absence of and in the presence of increased concentrations of MC-LR (0.01 nM to 100 nM). The excitation was set at 565 nm, and the emission spectra were collected from 570 nm to 750 nm. Excitation and emission slit widths were fixed at 5 nm and 5 nm, respectively. The fluorescence emission measurements were recorded according to Capo A. et al. [14]. All measurements were carried out at 25 °C, with an accuracy of ±0.5 °C, in PBS buffer, pH 7.4, in a total volume of 500 μL. The pre-incubation was performed at room temperature for 30 min. At the end of the pre-incubation step, steady-state spectra were acquired. The measurements were carried out in triplicate. For each measurement, the buffer contribution was subtracted from the experimental spectra, normalized by the maximum value. The data were analyzed with Origin Pro^®^ 2018 software.

### 2.8. Statistical Analysis

Fluorescence measurements and ELISA experiments were performed in triplicate; then, means and standard deviation (SD) were calculated. The fluorescence emission ratio was calculated by acceptor/donor maximum emission at 667 nm and 580 nm, and a linear regression fitting was applied to obtain a semilogarithmic calibration curve. The limit of detection (LoD) was calculated according to Shrivastava et al. [19] by the equation:

(6) $$\mathrm{LoD}=\frac{3.3\times S}{b},$$

where S is the standard deviation of the y-intercept of the linear regression, and b is the slope of the linear range. The graphs were realized by Excel 2016 Microsoft^®^ and/or by Origin Pro^®^ 8.0 software.

## 3. Results

### 3.1. Selection of Anti-Microcystin-LR and Characterization of the Binding Capability

The chemical structure of MC-LR is composed of a constant region of five amino acid residues and a variable region of two amino acid residues. In the present study, the variable region of the MC is composed of a leucine and an arginine. In order to select a specific monoclonal antibody anti-MC-LR, we focused our selection on a monoclonal antibody specific to an epitope of the variable region of the toxin. Even if this Ab recognizes all the 4-Arg microcystins, we decided to use it since it is the most toxic and abundant in freshwater.

**Figure 1:**
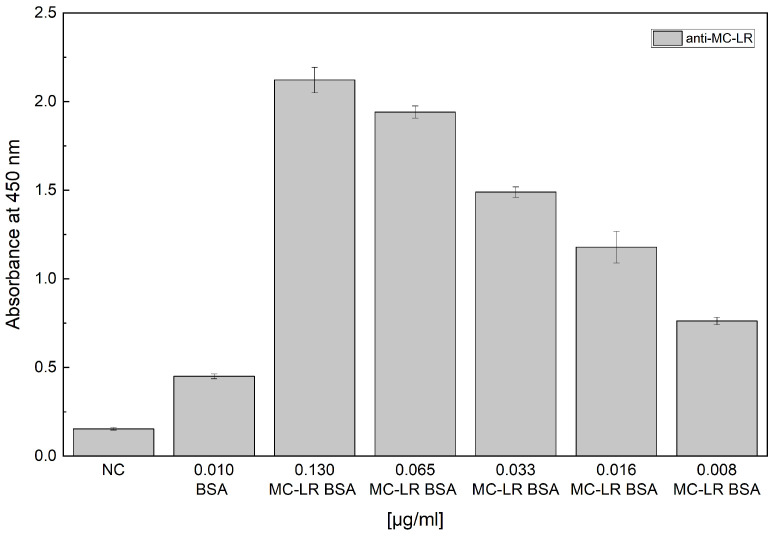
Evaluation of binding capability of unlabeled anti-MC-LR. Indirect ELISA test of unlabeled anti-MC-LR vs. MC-LR BSA. The experiments were performed in triplicate at 25 °C.

To evaluate the binding capability of the selected antibody, the MC-LR BSA conjugate was tested an indirect ELISA assay. Figure 1 reports the obtained results. The results indicate that the anti-MC-LR binds to MC-LR-BSA in a concentration range from 0.008 µg/mL to 0.13 µg/mL.

### 3.2. Production and Characterization of Anti-MC-LR 568 and MC-LR BSA 647

Anti-MC-LR 568 and MC-LR BSA 647 were characterized by SDS-PAGE, absorption, and fluorescence spectroscopy experiments.

SDS-PAGE analysis shows that anti-MC-LR 568 and MC-LR BSA 647 present a mobility shift with respect to the mobility of the unlabeled molecules, indicating an increase in the molecular weight associated with the presence of the fluorescence probe (Figure S1).

The absorption spectra analysis of anti-MC-LR 568 shows a red-shift from 562 nm (CF568 alone) to 565 nm (anti-MC-LR 568), while the absorption spectra analysis of MC-LR BSA 647 points out a blueshift from 652 nm (CF647 alone) to 650 nm (MC-LR BSA 647). The calculated DOL is 5.3 for anti-MC-LR 568. On the contrary, the DOL value for MC-LR BSA 647 is found to be 0.82. This is a lower value than the expected one (between 2 and 10), and it may be explained by the reduced number of reactive amino groups present on the MC-LR BSA complex with respect to BSA alone.

In Figure S2, we present the fluorescence emission spectra of anti-MC-LR 568 and MC-LR BSA 647. The maximum fluorescence emissions were observed at 580 nm for anti-MC-LR 568 and at 667 nm for MC-LR BSA 647.

To verify the binding capability of labeled anti-MC-LR 568, an indirect ELISA test was performed before and after the labeling procedure of anti-MC-LR.

**Figure 2:**
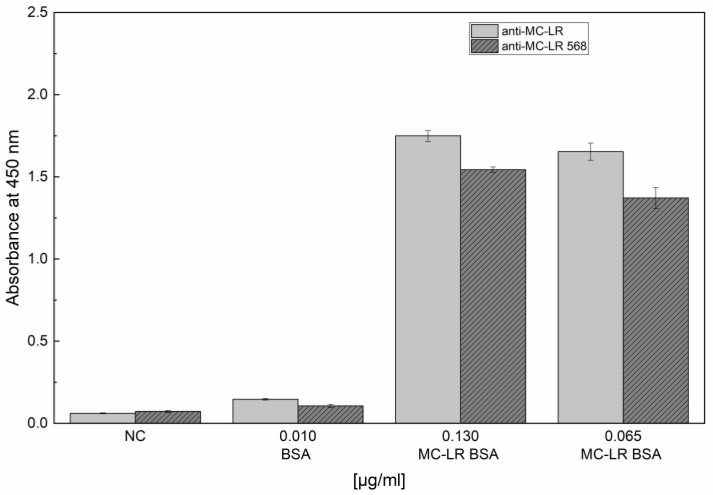
Evaluation and comparison of the binding capability of unlabeled anti-MC-LR and labeled anti-MC-LR 568. Indirect ELISA tests of unlabeled anti-MC-LR and labeled anti-MC-LR 568 vs. MC-LR BSA were performed. The experiments were performed in triplicate at 25 °C.

Figure 2 reports the obtained results. They confirm the capability of anti-MC-LR 568 to bind to MC-LR BSA.

### 3.3. FRET Assay Development

#### 3.3.1. Donor/Acceptor (D/A) Absorption and Emission Characterization

To develop the Förster energy transfer immunoassay, we selected Biotium CF568 and Biotium CF647 as a donor/acceptor (D/A) pair to label anti-MC-LR and MC-LR BSA.

**Figure 3:**
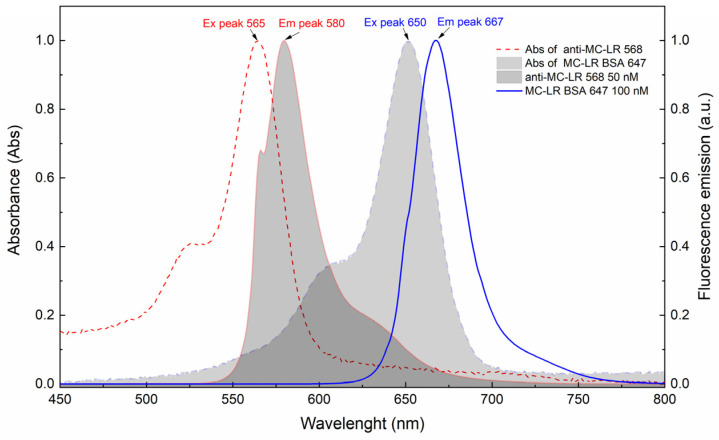
Spectroscopy characterization. Absorption and emission spectra of anti-MC-LR 568 and MC-LR BSA 647. The spectra were acquired at 25 °C. The fluorescence emission spectra were acquired upon excitation at 647 nm and 565 nm. All measurements were performed in 20 mM sodium phosphate buffer + 150 mM NaCl, pH 7.4. The spectra were normalized to 1.0. The temperature was set at 25 °C.

To evaluate the FRET efficiency, we compared the absorption and emission spectra of both anti-MC-LR 568 and MC-LR BSA 647. We observed a spectral overlap between the anti-MC-LR 568 emission curve and the MC-LR BSA 647 absorption curve (deep-grey region in Figure 3).

The FRET efficiency was calculated as a measure of energy transfer using the ratio between the intensity of the donor and acceptor (radiometric FRET) [16,20]. The ratio between the donor and acceptor depends on the fluorescence quantum yields of the two dyes. Radiometric FRET, given by the following equation, is usually a relative measure (E_rel_) of FRET.

(7) $$E_{\mathrm{rel}}=\frac{I_{A}}{{I_{D\;}+I}_{A}}$$

To evaluate the FRET efficiency, we performed titration experiments.

**Figure 4:**
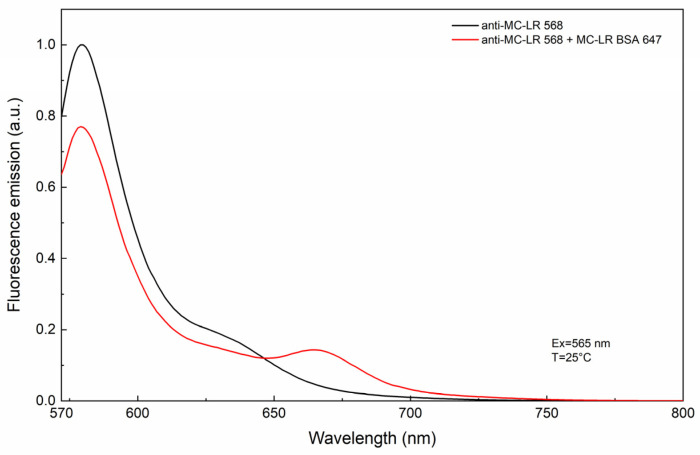
FRET efficiency evaluation. FRET efficiency evaluation via a titration experiment using anti-MC-LR 568 and increased concentrations of MC-LR BSA 647. The measurements were performed at 25 °C.

Figure 4 shows the fluorescence emission spectra of anti-MC-LR 568 in the absence of and in the presence of increased concentrations of MC-LR BSA 647.

By applying the equation reported above, we obtained a FRET efficiency value of 16%. The data also showed a reduction of the donor maximum intensity at 580 nm and an increase in the fluorescence intensity of the acceptor at 667 nm in the presence of 100 nM of MC-LR BSA 647. These data support the presence of energy transfer as a consequence of the interaction between anti-MC-LR 568 and MC-LR BSA 647.

#### 3.3.2. Microcystin-LR FRET Competitive Assay

A competitive assay was designed by using different donor/acceptor molar ratios.

We selected a donor/acceptor ratio of 1:2 with concentrations of 50 nM and 100 nM for anti-MC-LR 568 and MC-LR BSA 647, respectively.

**Figure 5:**
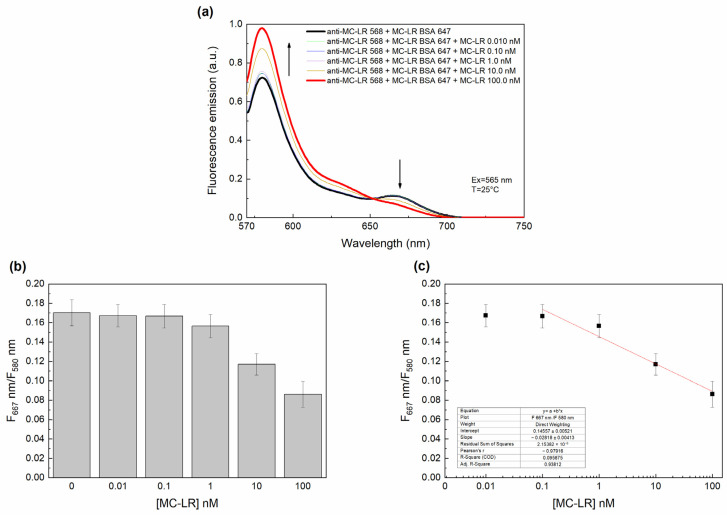
FRET Competitive assay. FRET competitive immune assay in the absence of and in the presence of unlabeled MC-LR (**a**). Ratiometric fluorescence emission (F_667_/F_580_) response in the presence of increasing concentrations of unlabeled MC-LR (**b**). The calibration curves of the assay were obtained through a linear fitting function (**c**). All measurements were acquired in PBS buffer, pH 7.4, upon excitation at 565 nm.

Figure 5a shows the results. Upon the increase in the MC-LR concentration, an increase in the donor fluorescence emission (peak at 580 nm) and a reduction of the acceptor fluorescence emission (peak at 667 nm) were observed. This indicates the displacement of MC-LR BSA 647 from the labeled antibody due to the presence of unlabeled MC-LR.

Figure 5b shows the ratio of the fluorescence emission acceptor/donor (F_667_/F_580_) plotted versus the MC-LR concentration values. A FRET signal reduction of 50% is observed in the presence of 100 nM of unlabeled MC-LR.

A linear correlation was observed in the range of 0.1 nM to 100 nM. Therefore, the calibration curve was calculated in the linear range to determine the detection limit (LoD) for the assay (Figure 5c). Each point represents the average of three experiments; the error bars represent the standard deviations of the mean. The LoD, calculated by 3.3 S/b, where S is the standard deviation of the y-intercept of the linear regression and b is the slope of the linear range, was estimated to be 0.245 nM (0.245 µg/L).

## 4. Conclusions

In this work, we proposed a suitable strategy by which to design and realize a competitive FRET assay to detect the presence of traces of MC-LR in water.

For this purpose, the following molecules were fluorescently labeled: (a) monoclonal antibody anti MC-LR was labeled with Biotium CF 568 (anti-MC-LR 568); (b) MC-LR BSA was labeled with Biotium CF 647 (MC-LR BSA 647). A solution of anti-MC-LR 568 was incubated with fixed amounts of the labeled MC-LR. Increasing amounts of unlabeled MC-LR were added to this solution. The variation in the FRET signal value was used to determine and quantify the presence of MC-LR. The assay showed an LoD value of 0.245 nM MC-LR (0.245 µg/L). This value is lower than the limit value as established by WHO.

## Data Availability

Data are contained within the article or Supplementary Materials.

## Supplementary Materials

The following supporting information can be downloaded at https://www.mdpi.com/1424-8220/24/10/3204/s1. Figure S1: SDS-PAGE analysis; Figure S2: Spectroscopic characterization of anti-MC-LR 568 and MC-LR-BSA 647.
