# Supplementary material for: A Förster Resonance Energy Transfer (FRET)-Based Immune Assay for the Detection of Microcystin-LR in Drinking Water
Source: Sensors (Basel). 2024 May 17;24(10):3204. doi: 10.3390/s24103204 (PMC11487384; doi:10.3390/s24103204)
Supplement: Supplementary file 1 [file sensors-24-03204-s001.zip › sensors-3013011-supplementary.pdf]

sensors

Article

A Förster Resonance Energy Transfer (FRET)-Based Immune Assay for the Detection of Microcystin-LR in Drinking Water

Alessandro Capo [1], Angela Pennacchio [2], Concetta Montagnese [2], Antonis Hadjiantonis [3], Panayiota Demosthenous [3], Alessandro Giusti [3], Maria Staiano [2], Sabato D'Auria [4,*] and Antonio Varriale [1]

1 Istituto di Scienze dell'Alimentazione, CNR, URT-Napoli, 80100 Napoli, Italy; alessandro.capo@cnr.it (A.C.); antonio.varriale@cnr.it (A.V.)
2 Istituto di Scienze dell'Alimentazione, CNR, 83100 Avellino, Italy; angela.pennacchio@cnr.it (A.P.); concetta.montagnese@cnr.it (C.M.); maria.staiano@cnr.it (M.S.)
3 CY.R.I.C Cyprus Research and Innovation Center Ltd., Egomi, 2414 Nicosia, Cyprus; antonish@cyric.eu (A.H.); p.demosthenous@cyric.eu (P.D.); alessandro@cyric.eu (A.G.)
4 Dipartimento di Scienze Bio-Agroalimentari, CNR, 00185 Roma, Italy
* Correspondence: sabato.dauria@cnr.it

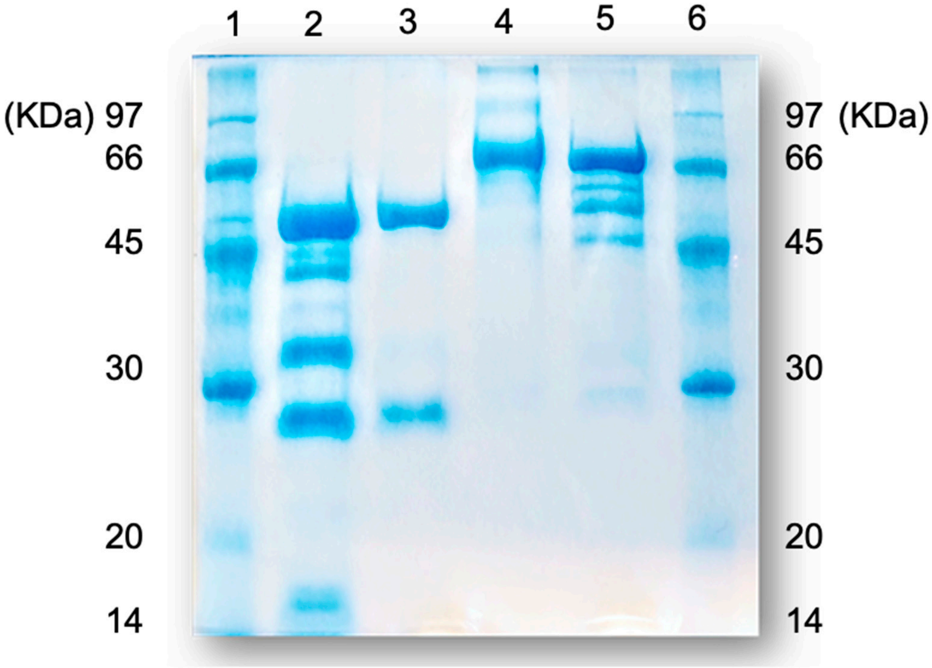

Figure S1: SDS-PAGE analsysis: 15% acrylamide SDS-PAGE standard molecular weight markers (line 1 and line 6); anti-MC-LR (line 2); anti-MC-LR 568 (line 3); MC-LR BSA 647 (line 4); MC-LR BSA (line 5).

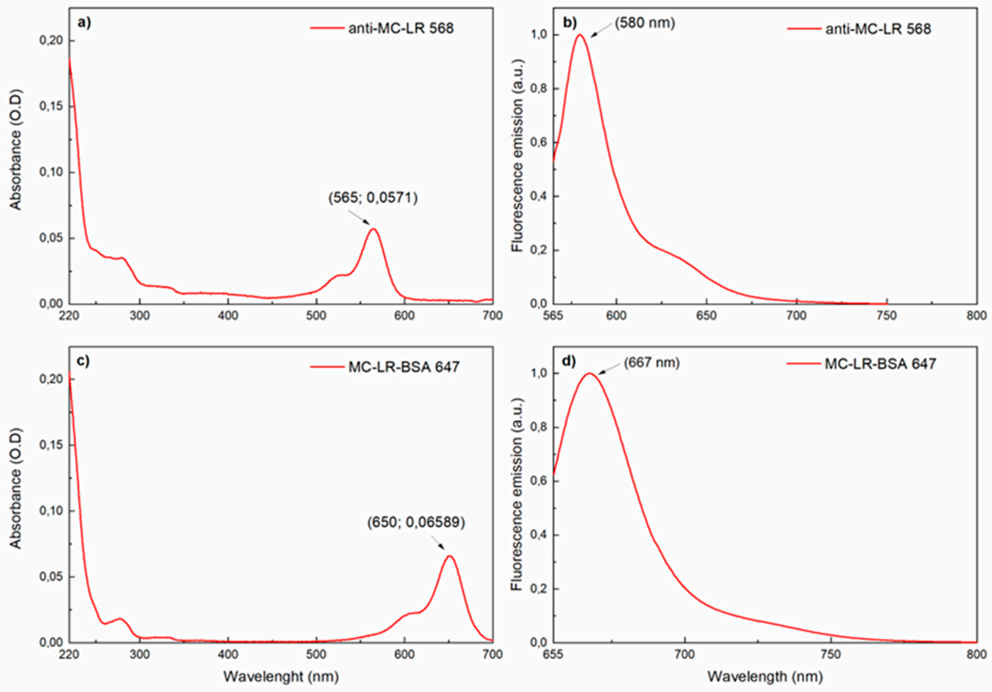

Figure S2: Spectroscopic characterization of anti-MC-LR 568 and MC-LR BSA 647: (a) absorption spectrum of anti-MC-LR 568; (b) fluorescence emission spectrum of anti-MC-LR 568 acquired upon excitation at 565 nm (temperature was set at 25°C); (c) absorption spectrum of MC-LR-BSA 647; d) fluorescence emission spectrum of MC-LR-BSA 647 acquired upon excitation at 650 nm (temperature was set at 25°C).
